# Influence Factors in the Wide Application of Alkali-Activated Materials: A Critical Review about Efflorescence

**DOI:** 10.3390/ma15186436

**Published:** 2022-09-16

**Authors:** Kaikang Liang, Kai Cui, Mohanad Muayad Sabri Sabri, Jiandong Huang

**Affiliations:** 1 Department of Architecture and Civil Engineering, City University of Hong Kong, Hong Kong 999077, China; 2 School of Civil Engineering, Dalian University of Technology, Dalian 116024, China; 3 Peter the Great St. Petersburg Polytechnic University, 195251 St. Petersburg, Russia; 4 School of Civil Engineering, Guangzhou University, Guangzhou 510006, China

**Keywords:** alkali-activated materials, alkaline cation leaching, carbonation, efflorescence

## Abstract

Applications related to alkali-activated materials (AAMs) have received much attention due to their excellent mechanical properties and low-energy production. Although much research has focused on developing AAMs, their application is still limited. One of the primary reasons is the efflorescence. Not only does efflorescence affect the material aesthetics, but it also affects the mechanical performance, leading to a decrease in material quality. This paper first summarizes the current research on AAMs efflorescence. The formation process of efflorescence is divided into three parts: alkaline cation leaching, air carbonation, and efflorescence formation. Furthermore, the influences caused by different factors, including raw materials, curing conditions, AAMs modalities, etc., on the efflorescence are proposed. This paper highlights the solutions for efflorescence by avoiding free alkaline cation leaching and preventing air carbonation. The advantages and disadvantages of efflorescence are discussed in-depth, showing that it can be exploited under certain conditions, such as in wastewater treatment. This paper has important implications for the practical preparation and application of AAMs.

## 1. Introduction

The commonly used material for concrete production is cement. However, cement production needs significant energy, leading to CO_2_ emissions [1,2]. As a kind of inorganic cementitious material prepared by activating raw materials rich in silica-alumina and calcium (fly ash (FA), ground blast furnace slag (GGBFS) and metakaolin (MK), etc.) by alkaline activators, such as sodium hydroxide (SH, NaOH), sodium silicate (SS, also known as water glass), AAMs have attracted wide attention for cement replacement because of their environmentally friendly production process [3,4] and excellent properties, such as high early-age strength [5], fire resistance [6] and fast hardening [7]. Although there have been many studies on preparing AAMs [8,9], there are still some problems before they can be widely applied. One of the critical issues is efflorescence.

**Figure 1:**
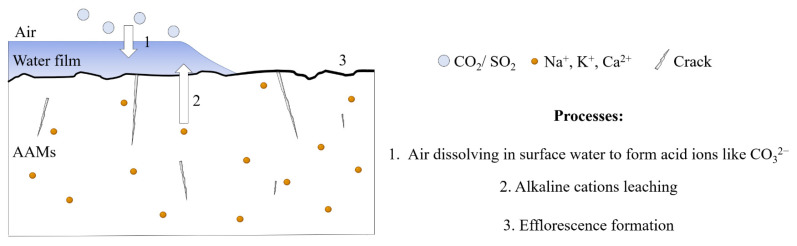
The efflorescence formation process.

Efflorescence is a common phenomenon in concrete applications. The efflorescence of cement-based materials mainly refers to CaCO_3_ formed by combining Ca(OH)_2_ and CO_3_^2−^ (CO_2_ in the free water). The free calcium hydroxide migrates through the hardened slurry to the material’s surface, which reacts with CO_2_ in the air to form calcium carbonate. Since there is no evidence that the Ca(OH)_2_ is the only product during the alkali-activated reaction, the efflorescence mechanism of the alkali-activated material is not the same as that of the cement-based material. The white efflorescence substances present in the alkali-activated materials might involve calcium hydroxide, calcium sulfate, sodium hydrate, potassium sulfate, sodium carbonate, potassium carbonate, and other substances, which are produced by the reaction between acid ions formed by the related gas dissolving in surface water and the free alkaline cations under a specific humidity condition, as shown in Figure 1 [10,11,12,13,14]. Compared to cement-based materials, AAMs easily form efflorescence because many alkaline cations exist in alkaline activators. Thus, the efflorescence formation equal to the loss of the raw material would significantly impact AAMs properties, especially alkali-activated foam concrete (AAFC) with severe efflorescence, due to the presence of many pores inside that provide channels for the movement of alkaline cations [15]. To further promote AAMs application in practice, there is a pressing need for efflorescence treatment.

This review provides a critical overview of the current studies on the AAMs efflorescence. We summarize the factors influencing efflorescence, including raw materials, cultivation conditions, AAMs modalities, etc. The impact on AAMs properties, especially mechanical properties, caused by efflorescence is discussed here. Based on the efflorescence formation mechanism, published studies on the solutions to deal with efflorescence are discussed from the following two aspects: avoiding the alkaline cations leaching process and reducing the CO_2_ adsorption (air carbonation). The shortcomings and possible improvements to these methods are also illustrated. Finally, this review proposes that the molecular dynamics (MD) simulation method provides a potential way to deeply understand and solve the efflorescence, allowing scholars to study efflorescence further. 

## 2. Investigations on the Influence Factors of Efflorescence

The possibility of efflorescence formation is related to the synthesis parameters, mainly involving the raw materials (solid aluminosilicate precursors, alkaline activators) [13,16,17], AAMs modalities, and the curing conditions [16,18].

### 2.1. Raw Materials

The raw materials for AAMs preparation contain materials rich in silica-alumina and alkali-activators. In particular, foaming agents are needed for AAFC preparation. The application of different raw materials may cause various effects on the efflorescence of AAMs.

#### 2.1.1. Solid Aluminosilicate Precursors

**Figure 2:**
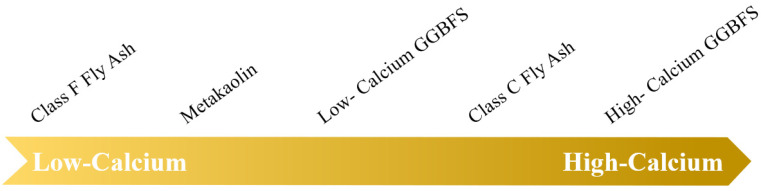
The Ca content level of different solid aluminosilicate precursors.

As the mechanism mentioned, the efflorescence of AAMs is related to the presence of Ca^2+^ and Na^+^. Na^+^ is mainly provided by the alkaline solution, which is described in Section 2.1.2. Solid aluminosilicate precursors mainly provide Ca^2+^. The Ca^2+^ content of different solid aluminosilicate precursors is shown in Figure 2. A study proposed that Ca^2+^ leaching (mass transport) was different, where 12 to 16 mol% of the total alkali content leaching was observed in FA-based AAMs [10], 17 to 30 mol% in MK-FA-based AAMs [13], and 40 to 60 mol% of the total alkali content in municipal solid waste incinerator FA-based AAMs [19].

In addition to the solid aluminosilicate precursors shown in Figure 2, there are some other industrial by-products (silica fume, steel slag, waste glass powder, etc.) used in AAMs production because they consist of silica, alumina, iron, magnesium, and calcium, which promote an overall reaction mechanism in alkaline environments [20]. Table 1 summarizes the current solid aluminosilicate precursors used for AAMs production; at the same time, the impact caused by these different precursors on efflorescence is also included. 

MK is a solid aluminosilicate precursor with low calcium content, which is obtained by calcination of natural kaolin at 650–800 °C [29]. Due to the high reactivity of metakaolin in alkaline environments, it is often used in studies of AAMs. Longhi et al. have carried out systematic studies on the efflorescence of metakaolin-based AAMs [13,30,31]. They pointed out that metakaolin-based AAMs usually had slight efflorescence due to their high reactivity [30]. The reasons for the severe efflorescence of these materials are more related to the alkaline activators’ content and the curing condition, which is discussed in the following sections.

FA is an industrial by-product resulting from the combustion of pulverized coal. The chemical composition, particle size, and reactivity of different types of FA vary depending on the coal source and combustion process [32,33]. ASTM C618-15 classifies FA into three classes based on chemical composition, Class N, Class F, and Class C [34]. Class F FA has only zeolitic properties and a low calcium oxide content, while Class C FA has higher calcium oxide and cementitious properties. Class N FA consists of natural zeolite in its raw or calcined form. Class F FA is more frequently used in studies, but Class C is also often used [35]. Except for the Ca content, the liquid/solid ratio FA required for AAM production has an essential effect on efflorescence. The FA with a high liquid/solid ratio requirement demonstrates rapid efflorescence [11]. Moreover, the Si/Al ratio of FA also effectively affects efflorescence. Wang et al. [22] pointed out that the lowest level of efflorescence under the Si/Al ratio is 1.5 due to the content of [AlO_4_]^−^ structures, which were highest under this ratio, leading to a relatively small pore volume and size. The dense matrix and [AlO_4_]^−^ structures of AAMs help reduce the efflorescence degree significantly. 

GGBFS is the residual material from metal refineries. Slag-based AAMs are praised for their low energy consumption, low carbon emission, and outstanding durability. The composition of GGBFS is close to that of cement, which contains CaO, MgO, SiO_2_, and other components. The usage of GGBFS for the preparation of AAMs is associated with more severe alkaline cation leaching due to its higher CaO content than other raw materials [36]. However, the composite usage of GGBFS with other raw materials has been reported to enhance the material strength, leading to a lower defect, which, on the other hand, would reduce alkaline cation leaching [37].

Further studies are needed on the optimal GGBFS content and corresponding effects on efflorescence. Other materials, such as palm oil fuel ash (POFA), a kind of primary agricultural waste, have also been proposed to result in efflorescence. This material is rich in silica and calcium and has recently been tested as a source of aluminosilicate [38,39]. So far, it has been used as part of binary and ternary mixtures with other conventional POFAs for producing AAMs.

#### 2.1.2. Alkaline Activators

Alkaline activators are essential for AAMs preparation because they initiate chemical processes and react with alumina, silica, and other compounds in solution. Commonly used activators involve SH, SS, potassium hydroxide (KOH), and calcium hydroxide (Ca(OH)_2_). These alkaline activators contain many hydroxyl ions (OH^−^), which help dissolve aluminates and silicates present in alkali-activated precursors. At the same time, these compounds also contain many alkaline cations, which influence efflorescence. Ismail et al. [40] used SH and SS to activate FA-slag-based AAMs. FA was mainly activated with SH. Other additives can be added for further activation [35]. Novais et al. [41] used SH and SS solutions to activate FA and MK-based alkali-activated material. Suh et al. [42] activated FA-based materials with solutions containing Ca(OH)_2_ and Na_2_CO_3_. Efflorescence in AAMs usually occurs when there is an excess of unreacted Na^+^/Ca^2+^ in the mixture [10,43]. These leaching alkaline cations become mobile in the pore network due to the flow of water in the mix. Subsequently, alkaline cations come into contact with CO_2_ from the surrounding atmosphere to form white substances [16,43]. Generally, AAMs with high sodium concentrations are more prone to efflorescence, depending on the type of activator used [44]. SS and SH increase the overall sodium content, leading to higher efflorescence in AAMs [44,45]. It was reported that potassium-based activators develop less efflorescence in AAMs than sodium-based activators. This can be attributed to potassium’s higher reactivity than sodium. The atomic radius of potassium is greater than that of sodium, leading to the outermost electron being more easily lost than in sodium. Mixes activated with a potassium-based solution easily combine with K^+^, which shows that they contain lower contents of unreacted or leached free alkaline cations [46]. Puertas et al. [47] found that the Na^+^ concentration in AAMs pore solutions activated by SS exceeded 1200 mmol/L. When some transport forces, such as gravity, capillary action, etc., exist, free Na^+^ continuously migrates to the surface, resulting in severe efflorescence. In contrast to what we inferred before, the usage of Ca(OH)_2_ can reduce the alkali diffusion coefficients of AAMs by up to an order of magnitude, even when Ca^2+^ is added [48]. Zhu et al. [49] concluded that the addition of Ca(OH)_2_ accelerated the reaction process of AAMs at an early age, so the early compressive strength of the material significantly increased. In addition, the percentage of mesopores decreased after adding Ca(OH)_2_, which leads to the introduction of calcium, which is supposed to inhibit efflorescence in AAMs by effectively enhancing their permeability. 

### 2.2. AAMs Modalities

According to the production methods, the AAMs modalities involve conventional AAMs (alkali-activated mortar, alkali-activated concrete) and AAFC. Compared to conventional AAMs, AAFC has a higher possibility for efflorescence due to its porous structure, which provides channels for alkaline cation transportation. A critical step of AAFC production is the production of bubbles inside the material. Commonly used foaming methods involve physical and chemical methods. The chemical foaming method refers to adding chemical reagents and catalysts into the pure slurry to generate oxygen or hydrogen by a chemical reaction at a specific temperature. Al powders are commonly used for bubble generation. The mechanism of Al usage for bubble generation is shown as Equations (1) and (2).

(1) $$2\mathrm{Al}+6H_{2}O=2\mathrm{Al}{\left(\mathrm{OH}\right)}_{3}+3H_{2}$$

(2) $$\mathrm{Al}{\left(\mathrm{OH}\right)}_{3}+\mathrm{NaOH}={\mathrm{NaAlO}}_{2}+2H_{2}O$$

As shown in Equation (2), the formation of AlO_2_^+^ could combine with Na^+^, which provides a weak binding force to avoid Na^+^ transportation. However, it has been found that the chemical foaming method is unstable because the foaming rate and foam quality are strongly uncontrollable, resulting in significant differences among the sizes and uniformities of pores [50]. Instead of Al powder, the H_2_O_2_ shows accelerated decomposition with increased curing temperature. If the curing temperature is too high, the sample becomes loose, and the matrix becomes fragmented. As a result, the porosity and bulk density increases significantly while the compressive strength decreases, which leads to severe efflorescence. The physical foaming method refers to forming prefabricated bubbles by a foaming agent, which is added to the pure slurry by mechanical mixing. Compared with the chemical foaming method, bubbles prepared by the physical foaming method are not easily influenced by the corresponding environments. The state of the water pore system is relatively more stable [51]. Therefore, the physical foaming method has become a fairly popular method for producing FC. In addition to physical and chemical foaming methods, light aggregates, such as expanded polystyrene (EPS), are used to prepare AAFC. Prasittisopin et al. [52] proposed that adding EPS may cause delamination due to the opposing bonding forces between the EPS and the slurry, which leads to higher porosity and defects that are not conducive to efflorescence treatment.

### 2.3. Curing Conditions

Usually, curing conditions for AAMs production mainly involve ambient, thermal, water, standard, and overlay curing [53]. The efflorescence of AAMs is primarily due to the migration of Na^+^ and Ca^2+^ from inside to the surface, forming salt compounds with CO_3_^2−^, HCO_3_^−^, etc. The alkaline cation migration process and air dissolving process are caused by the moisture difference between the inner and outer surfaces of the material. Different curing conditions provide AAMs with varying moisture conditions, resulting in different efflorescence rates.

Ambient curing is always thought of, in practice, as a process that requires minimal effort and almost no additional cost [54]. Although AAMs cured at ambient temperatures (usually between 20 and 30 °C, with generally exercised relative humidity (RH) of 40–95%) are convenient for engineering applications, materials quickly lose/adsorb water under this condition. The difference in humidity inside and outside the material leads to the migration of free water, which brings the free alkaline cations to the surface, where they react with CO_3_^2−^, HCO_3_^2−^, etc. When the external humidity is lower than the humidity inside the material, the cations migrate towards the surface through the evaporation of water. On the other hand, CO_2_ storage is enhanced when the external humidity is above 90%, and CO_2_ reacts with OH^−^ in the pore solution to form CO_3_^2−^/HCO_3_^2−^ through capillary water absorption. Next, when the concentration is supersaturated, alkaline cations combine with CO_3_^2−^/HCO_3_^2−^ to form an apparent solid carbonate [55]. Moreover, as reported in [56], ambient curing also leads to incomplete alkali-activated reactions, resulting in the leaching of alkaline cations, and promoting crystallization formation on the concrete surface [57,58].

Thermal curing is the most common method for low reactive binder curing, which includes low calcium-based AAMs. Studies have shown that heating improves materials’ early strength and reduces porosity [59,60]. The reason is that the alkali-activated reaction is promoted, leading to early hardening state properties. Although thermal curing could help with efflorescence treatment, a suitable curing time is needed. It has been shown that the temperature range of the first 24 h for low-calcium-based AAMs and the first 6 h for high-calcium-based AAMs plays a crucial role in the strength improvement of AAMs [61]. Long-term thermal curing (aging) under dry conditions leads to higher dry shrinkage cracking and porosity, which is not beneficial for efflorescence treatment. According to the literature, while thermal curing is appropriate for depleting the sodium content and reducing the total porosity of AAMs [16], proper humidity in the environment surrounding the specimen is a more straightforward method to reduce efflorescence significantly [62].

**Figure 3:**
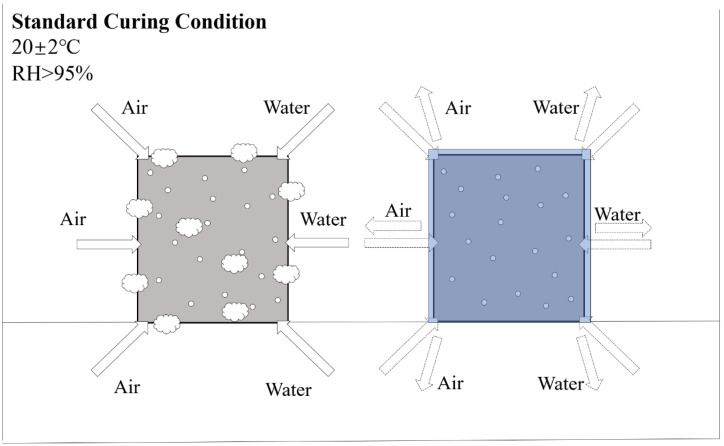
The positive effect on efflorescence is caused by overlay curing (20 ± 2 °C, RH > 95%).

Water curing refers to immersing the specimens in water at ambient temperatures (20–25 °C) or hot water (65–95 °C) [63]. Different efflorescence caused by water curing is mainly related to the compositional content of AAMs [64,65]. For low calcium-based systems, water curing could cause material strength reduction and alkaline cation leaching [66]. In high-calcium-based systems, water immersion can have the opposite effect, resulting in high compressive strength [67] and lower permeability [68]. The difference is mainly caused by the rapid formation of the silica-rich matrix, including calcium silicate hydrate (C-S-H) in the pore space within high calcium mixtures through water consumption, which contributes to the early strength formation [69,70,71]. It is worth noting that water curing is not commonly used for AAFC curing; specimens are usually placed in the standard curing conditions for replacement. Due to the high levels of soluble alkali metals (i.e., Na^+^ ions) in alkali-activated systems, the presence of exudate is an obvious problem when AAFC is exposed to excess moisture provided by the standard curing room [72]. For AAFC with low densities (under 500 kg/m^3^), the pores easily adsorb a large amount of water and CO_2_, leading to serious efflorescence. Thus, specimens should be cured with overlay, which could effectively avoid the transportation of alkaline cations and CO_2_, as shown in Figure 3. Zhang et al. [10] proposed the idea that, contrary to the usual understanding, porous structures provide more channels for the transport of water and base cations. They attributed the reason to the fact that the pores of low-density AAFC are too large for efficient capillary transport of water. In this case, the carbonate crystals grow inside the material, leading to sub-efflorescence rather than on the surface, the effects of which should be further investigated.

## 3. Impact on the Properties

**Figure 4:**
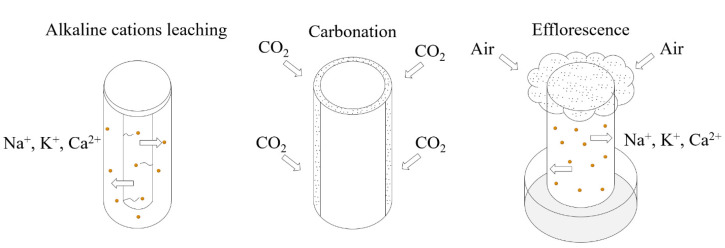
The testing methods of alkaline cation leaching, air carbonation, and efflorescence.

Technically, efflorescence formation involves three steps: alkaline cations leaching, CO_2_ dissolving in surface water, and efflorescence. All three steps affect AAMs’ properties [13]. The leaching condition is developed by the complete immersion of AAMs in deionized water, with a mass proportion of 1/20 (geopolymer/water). The development of CO_2_ dissolving can cause natural air carbonation to happen naturally. AAMs were exposed to efflorescence by the partial immersion of the AAMs (to ~5 mm depth) in distilled water, with the remainder of the sample open in ambient conditions (25 ± 5 °C and RH = 65 ± 15%), as shown in Figure 4.

Alkaline cation leaching is the first process related to efflorescence formation, which causes the removal of free or weakly bonded alkaline cations, resulting in an incomplete alkali-activated reaction. As proposed in [11], after 28 days of curing, the compressive strengths of AAMs in the air increased by 20–35%, compared to their demolding strengths, while the samples fully immersed in deionized water showed much lower increases in strength. The difference is mainly due to the lower reaction extent caused by the alkaline cation loss. The flexural and tensile strengths of the AAMs are also sensitive to exposure to leaching conditions. The leaching process induces the reduction in Q_4_(4Al) and Q_4_(3Al) silicate sites within the geopolymeric matrix, indicating a structural change due to the alkaline cation removal, with some instability of sodium aluminosilicate gel under leaching [13]. 

Exposure to air carbonation conditions induced a carbonation process in the first layer of AAMs related to efflorescence formation. The reaction mechanisms of AAMs in calcium-rich and low-calcium systems are different. In the calcium-rich AAM system, CO_2_ dissolves into the pore solution to form carbonic acid and directly reacts with C-S-H/calcium-aluminate-silicate-hydrate (C-A-S-H) gel to form CaCO_3_. The decalcification of C-S-H/C-A-S-H provides the Ca^2+^. Due to decalcification, the C-A-S-H gel undergoes volume shrinkage and an increased degree of polymerization.

Further carbonation transforms the gel into a silica gel, resulting in greater shrinkage. Carbonation causes significant shrinkage of materials, resulting in microcracks around the aggregate, increasing the porosity and the CO_2_ diffusion coefficient of the AAMs. Mei et al. proposed a similar conclusion. They pointed out that AAMs’ compressive strength decreases dramatically during CO_2_ attacks, with the most rapid strength loss occurring during the first 3 days. After 7 days, the structure of the AAMs samples collapsed. The damage to the AAMs was mainly caused by the conversion of Ca^2+^ in the C-S-H/C-A-S-H phase of the matrix into crystalline carbonates after a CO_2_ attack, which leads to interfacial bonding force weaking and a sharp increase in matrix pores. These cracks and holes further provide paths for CO_2_ attacks, thereby accelerating the carbonization of AAMs. Carbonation is divided into two steps for the low-calcium AAMs system, as proposed in [73]. First, during the first two weeks, the nearly complete carbonation of the pore solution happens with Na_2_CO_3_ formation. Secondly, the carbonate/bicarbonate phase equilibrium results in bicarbonate formation. The study proposed that natrium-aluminate-silicate-hydrate (N-A-S-H) is stable during carbonation, unless OH^−^ from the pore solution reacts with carbonic acid [74]. Based on the discussion about carbonation in a low-calcium AAM system and N-A-S-H stability, with prolonged use, NAHCO_3_ will consume OH^−^ to form NA_2_CO_3_, and such a transformation is not conducive to the stability of the N-A-S-H structure. The formation of efflorescence involves the above two steps. Besides the effect mentioned before, Yao et al. [75] pointed out that efflorescence formation induces carbonate crystallization, and internal stress can develop, contributing to a possible microstructure damage tendency. 

## 4. Solution Methods

The essence of the alkaline cation leaching phenomenon is that Ca^2+^ and Na^+^ inside the material migrate to the surface of the material with moisture, and CO_2_ in the air dissolves into the surface water to form carbonate/bicarbonate and combines with Ca^2+^ and Na^+^ to form a white powder-like object that remains on the surface of the material. Thus, the way to solve this problem must also start at the mechanism level. The first step is to avoid the leaching of free alkaline cations. The second is to reduce the CO_2_ adsorption inside the material.

### 4.1. Prevention of Alkaline Cation Leaching

Najafi et al. [16] observed that the most effective methods for avoiding alkaline cation leaching included the addition of alumina-rich additives to the powder and hydrothermal curing, both of which helped to improve the alkali retention in the AAMs. The addition of alumina reduced alkali mobility due to the increase in AlO_4_ sites within the M-A-S(-H) gel framework, while hydrothermal curing increased the extent of the reaction and the amount of M-A-S(-H) gel formation, reducing porosity. Therefore, the best strategy to reduce the leaching of free alkaline cations involves promoting the alkali-activated reaction, increasing the material’s compactness, and preventing water fluidity [23,25].

#### 4.1.1. Promotion of the Alkali-Activated Reaction

The activity of the raw material can be stimulated by different methods, including grinding [76], etc., to promote the alkali-activated reaction [77]. In addition, higher soluble silica contents in the activator increase the degree of reaction and gel formation [48]. Longhi et al. [13] gave similar conclusions, where the addition of soluble silica to the activator increased the degree of gel formation and the Si/Al ratio in the gel framework. The addition of calcium aluminate cement (CAC) as an additional source of calcium and alumina was also effective in reducing efflorescence because the added material allows more Na^+^ binding at the charge balance point in M-A-S(-H) gels, as previously reported [16]. Rice husk ash (RHA) is a major agricultural by-product with processing challenges, including high silica content, and has been investigated as a precursor to AAMs. It can have good boiling properties and has been used to make special concrete [78,79]. RHA used in AAMs has been found to affect the heat of hydration of mixtures and improve the alkali-activated reaction rate, resulting in less alkaline cation leaching [78]. 

#### 4.1.2. Increase in Material Compactness

We usually consider the improvement of material strength to be related to increased material compactness. It is generally assumed that adding slag to other precursors can make the system more compact and reduce the possibility of Na^+^ or Ca^2+^ migration. The reason is that adding slag to AAMs can reduce the average pore size and total porosity [10,80], thereby increasing the mechanical strength [81]. Slag can also prevent the incorporation of bubbles in AAMs and reduce the formation of large pores [82]. These effects are also thought to result from the high calcium content in the slag, leading to calcium aluminate hydrate (C-A-S-H) gels forming in the N-A-S-H system. However, some studies have taken the opposite opinion. They pointed out that the replacement of metakaolin with FA and GGBFS does not reduce efflorescence due to the reduction in the reaction range [31]. At the same time, the high calcium content of slag would lead to an increase in alkaline cation leaching possibility. Superfine FA, a microsphere particle with a good ball effect, can effectively adjust the working performance of AAMs [83]. The particle size of superfine FA is comparable to that of AAMs, and it does not have a solid water-snatching effect, which ensures the fluidity of AAMs. Moreover, the usage of superfine FA could produce hydrated calcium silicate gel that helps fill the connected pores to increase AAMs’ compactness. A hybrid solution with SS should be used to obtain sufficient strength early in the curing process, which is another compact structure production method [84]. As reported by [85], replacing the SS solution with SF produces high-strength concrete compared to the widely used SS or the SS and SH combination solutions. 

The development of nanotechnology significantly impacts materials, manufacturing, and other fields. Nanomaterials have been widely used as additives in material modifications [86]. The effects caused by nanomaterial addition mainly involve the following three aspects: the nucleation effect, fine particle filling effect, and high pozzolanic activity. As mentioned before, nano-silica and nano SiO_2_ are proposed as additives for AAM production. In addition, nano-alumina, nano-CaCO_3,_ and nano-TiO_2_ are also regarded as potential additives [83,87,88]. These nanomaterials produced by the sol-gel method have small particle sizes, narrow particle size distribution, and spherical particles. The nucleation effect and micro aggregate filling effect caused by nanomaterial addition are favorable for improving material compactness. However, not all nanomaterial additives result in a denser structure of the AAMs. The addition of nano-silica has been regarded as detrimental to the compactness of the internal structure and to the material’s permeability [89]. Since there are various methods of material strength enhancement, to reduce the complexity of experimental testing in advance, Artificial Intelligence can be used to predict the expected enhancement results contributing to the more effective enhancement method selection [90,91].

### 4.2. Reduction in the CO_2_ Adsorption

Carbonation is an essential indicator of CO_2_ entering the material. Various studies have reported that the addition of calcined hydrotalcite (C-HT), calcium silicate (CS), and Gypsum could help prevent CO_2_ from entering the material [92]. Ke et al. [93] demonstrated the superior ability of C-HT to fix CO_3_^2−^ in alkali-activated slag cement. Shui et al. [94] reported that adding C-HT effectively improved the resistance to carbonation. The improvement in carbonation resistance by adding C-HT should be attributed to the interlayer anion exchangeability [95] and the structural reconstruction memory effect [96], where the CO_2_ invaded by C-HT is immobilized to avoid the depletion of alkaline pore solutions in the matrix to some extent. In addition to C-HT, it has been reported that CO_2_ can react with C.S. and water to precipitate calcium carbonate and amorphous silica [97], reducing the alkalinity loss. However, there is no direct evidence to confirm this approach’s effectiveness. Gypsum can react with aluminates in AAMs to form Al_2_O_3_-Fe_2_O_3_-mono (Afm) or Al_2_O_3_-Fe_2_O_3_-tri (Aft) [98], which improves the impermeability of AAMs and increases the diffusion resistance of CO_2_. Besides improving impermeability, carbonation can also be prevented by reducing the free water on the material surface, which can be achieved by adding some water-repellent materials, such as calcium stearate [25]. Since calcium stearate has low surface energy with superhydrophobic properties, it reduces the contact angle of water and resists water diffusion into the geopolymer matrix, preventing CO_2_ from dissolving in water.

## 5. Prospects and Challenges

### 5.1. Wide Practical Application

**Figure 5:**
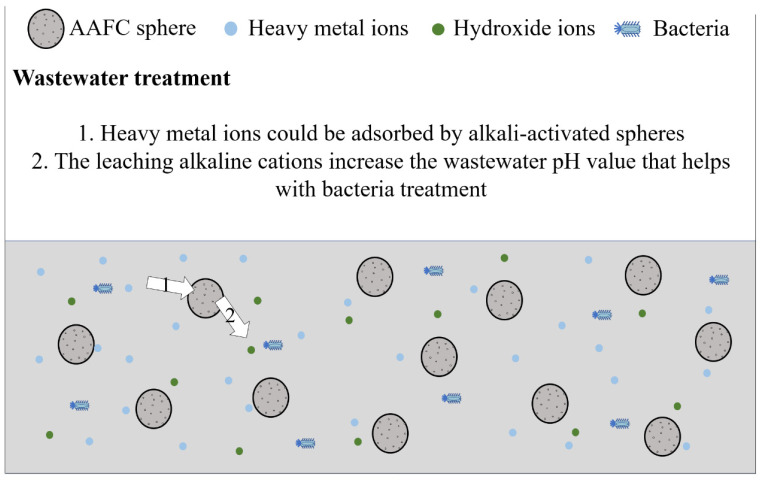
The prospects of using the AAFC sphere for wastewater treatment.

Compared to cement-based materials, the usage of AAMs needs to be further improved. For conventional AAMs, especially AAFC used as external insulation wall panels, materials are easily exposed to the external environment, where the temperature and humidity differ from indoors. Thus, external insulation wall panels are more prone to efflorescence than indoor applications. The effective methods for efflorescence treatment can promote a complete alkali-activated reaction, which is beneficial for solving the problems of chalking and peeling of the material due to the loss of strength caused by efflorescence. Efflorescence does not always result in a negative impact on the AAM application. The application conditions of AAMs could be widely increased if efflorescence is used effectively. AAFC has been proposed as a potential heavy metal ions adsorbent due to the presence of many active binding sites provided by the many pores in the material. Tan et al. [99] proposed that the production of the AAFC sphere could be a better modality for wastewater treatment due to its facilitative recyclability. Under this condition, efflorescence might have a positive effect, including the combination of heavy metal ions and the leached hydroxide ions and the bacteria treatment due to the increasing pH value caused by the efflorescence items and hydroxide ions, as shown in Figure 5. These positive effects should be studied further.

### 5.2. Challenges in Composite Mix Ratio

As mentioned before, whether it is by Si/Al ratio adjustment or by the addition of materials to promote the complete alkali activation reaction, all those changes are related to adjustment of the mix ratio. According to the studies mentioned before, it is known that several methods (appropriate Si/Al ratio, the mixture of SH and Na_2_SiO_3_, the usage of Ca(OH)_2_, the use of composite raw materials (FA, slag combined), the addition of nanomaterials, etc.) can effectively reduce the efflorescence. However, no studies can systematically summarize and quantify a suitable ratio from multiple perspectives using experimental or simulation methods. In addition, it is stated in most studies that the movement of alkaline cations is due to capillary action, along with free water. It is uncertain whether some forces would have an adsorption effect on alkaline cations or whether different external environments would affect the movement of alkaline cations. The movement of alkaline cations in materials can be reviewed using molecular dynamic (MD) simulation methods, which could reflect the forces inside. The MD simulation method is powerful for understanding targeted materials’ molecular and atomistic data [100,101]. Some intricate material characteristics that cannot be fully explored experimentally, such as the ion movement, the H-bonding generation, and the monitored reaction process of the targeted molecule, could be further studied using the MD simulation method.

## 6. Conclusions

Due to their low production cost and excellent performance, AAMs have been studied for several years and applied in different engineering conditions, including road filling, external thermal walls, etc. Although efflorescence is a common phenomenon in concrete usage, compared to OPC materials, AAMs more easily form efflorescence because the alkaline activators contain many alkaline cations. The formation of efflorescence also has a more severe effect on AAMs’ properties. This paper reviews the experimental studies related to Efflorescence of AAMs. It summarizes the impact on Efflorescence of AAMs caused by different factors, including raw materials, AAMs modalities, and curing conditions. Based on the summary, to reduce the efflorescence, we prefer to use aluminosilicate precursors with low calcium content, such as metakaolin and Class F FA, to be activated by KOH or Ca(OH)_2_. The denser AAMs with fewer pores could be cured under thermal curing conditions, which helps to promote the alkali-activated reaction. Loose AAMs with many pores inside should be cured to reduce the alkali cations and air transportation. In addition, nanomaterials, such as nano-SiO_2_, are potential additives that may enhance the compactness of the material’s structure. The efflorescence phenomenon can also be an advantage under some specific applications. AAFC is considered a potential heavy metal treatment material due to the presence of many pores inside the material that can provide active binding sites for heavy metal ion adsorption. The unreacted hydroxide ions can combine with heavy metal ions in the effluent to form alkaline precipitates. In addition, the leaching alkali can also regulate the pH of the wastewater, and the rise in the pH is conducive to the destruction of microorganisms and bacteria. MD simulation is a potential method for deeply understanding ion transportation and efflorescence formation; it could also be an effective and economical way to explore the suitable mix ratio of AAMs with minimal efflorescence. The related aspects of the study deserve further exploration.

## Figures and Tables

**Table 1:** The efflorescence degree of AAMs produced under different conditions.

Solid Aluminosilicate Precursors	Activator SiO_2_/Na_2_O Molar Ratio	Total Na/Al Molar Ratio	Si/Al Ratio	Solution/Solid Ratio	Additive	Curing Condition	Efflorescence Degree	Reference
Natural pozzolan	0.45	0.61	None	None	High-alumina cements Secar 71	25 °C RH95%	1.26%	[16]
Waste glass powder Class C FA Limestone	None	None	None	0.4	None	20 °C Air curing	2.8%	[21]
Class F FA Bauxite	None	None	1.5	None	None	80 °C oven	None	[22]
FA	1.5	None	None	None	None	25 ± 1 °C RH 90 ± 10%	4.0%	[11]
FA GGBFS	1.5	None	None	None	None	80 °C Hydrothermal	16.0%	[10]
Class F FA	1.2	None	None	None	Octyltriethoxysilane	20 °C RH > 95%	3.5%	[23]
Class F FA GGBFS	2.0	None	None	None	Fumed silica	None	None	[24]
FA	None	None	None	None	5% calcium stearate	65 °C	0.0%	[25]
Construction and demolition waste	0.67	None	None	None	20 wt% MK	None	10.2%	[26]
Steel slag and blast furnace slag	None	None	None	None	15% 5A zeolite	20 °C RH > 95%	None	[27]
Steel slag and blast furnace slag	None	None	None	None	2.0% nano-silica	20 °C RH > 95%	None	[28]
Steel slag and blast furnace slag	1.4	None	None	None	3.0% nano-SiO_2_	20 °C RH > 95%	None	[14]
